# An approach to improve the performance of subject-independent BCIs-based on motor imagery allocating subjects by gender

**DOI:** 10.1186/1475-925X-13-158

**Published:** 2014-12-04

**Authors:** Jessica Cantillo-Negrete, Josefina Gutierrez-Martinez, Ruben I Carino-Escobar, Paul Carrillo-Mora, David Elias-Vinas

**Affiliations:** Subdirection of Technological Research, Instituto Nacional de Rehabilitación, Mexico City, 14389 Mexico; Department of Electrical Engineering, Centro de Investigación y de Estudios Avanzados del IPN, Mexico City, 07360 Mexico; Division of Neuroscience, Instituto Nacional de Rehabilitación, Mexico City, 14389 Mexico

**Keywords:** Brain computer interfaces, Event related Desynchronization, Common spatial patterns, Linear Discriminant analysis, Stroke

## Abstract

**Background:**

One of the difficulties for the implementation of Brain-Computer Interface (BCI) systems for motor impaired patients is the time consumed in the system design process, since patients do not have the adequate physical nor psychological conditions to complete the process. For this reason most of BCIs are designed in a subject-dependent approach using data of healthy subjects. The developing of subject-independent systems is an option to decrease the required training sessions to design a BCI with patient functionality. This paper presents a proof-of-concept study to evaluate subject-independent system based on hand motor imagery taking gender into account.

**Methods:**

Subject-Independent BCIs are proposed using Common Spatial Patterns and log variance features of two groups of healthy subjects; one of the groups was composed by people of male gender and the other one by people of female gender. The performance of the developed gender-specific BCI designs was evaluated with respect to a subject-independent BCI designed without taking gender into account, and afterwards its performance was evaluated with data of two healthy subjects that were not included in the initial sample. As an additional test to probe the potential use for subcortical stroke patients we applied the methodology to two patients with right hand weakness. *T*-test was employed to determine the significance of the difference between traditional approach and the proposed gender-specific approach.

**Results:**

For most of the tested conditions, the gender-specific BCIs have a statistically significant better performance than those that did not take gender into account. It was also observed that with a BCI designed with log-variance features in the alpha and beta band of healthy subjects’ data, it was possible to classify hand motor imagery of subcortical stroke patients above the practical level of chance.

**Conclusions:**

A larger subjects’ sample test may be necessary to improve the performances of the gender-specific BCIs and to further test this methodology on different patients. The reduction of complexity in the implementation of BCI systems could bring these systems closer to applications such as controlling devices for the motor rehabilitation of stroke patients, and therefore, contribute to a more effective neurological rehabilitation.

## Background

Brain Computer Interfaces (BCIs) decode a subject’s intention from EEG signals and translate it into control signals for an external device, providing a new communication channel without using traditional pathways (nerves and muscles). BCIs have great potential as a tool for aiding patients with motor disabilities due to a brain injury, as stroke. Nowadays, despite BCI systems based on motor imagery (MI) are being examined as a neural rehabilitation device, they are designed and tested exclusively in research centers, and most of them analyze only data from healthy subjects [1–4]. One of the biggest challenges that BCIs face, in order to be used in the clinical field, is to reduce the time spent on its design which includes: EEG signal acquisition, feature extraction, classification and system calibration [5]. The classifier training stage requires a significant number of EEG signal features to be able to recognize MI patterns of a specific subject with an acceptable performance, which requires long recording sessions and afterwards several training sessions in order to the subject to be able to use the system. This type of design is known as subject-dependent BCI (SD-BCI) [6, 7]. However, it is common that some patients, e.g. stroke patients, do not have the physical or mental availability to complete hundreds of trials required for the SD-BCI implementation. The design of systems that enable a new user to achieve good performance with minimal training could decrease the tiresome sessions. Consequently, to achieve that goal, systems trained with the data of a group of subjects instead of a single subject have been developed, these systems are known as subject-independent BCI (SI-BCI) [8, 9].

Most of the research groups have focused their work in SD-BCIs based on the fact that there is a large variability of EEG signals between subjects, but considering the hypothesis that states that it is possible to find common information in the EEG data of some subjects, SI-BCIs have gained interest in the international scientific community due to the advantages that a minimal training offers. As an example, the authors of [8] explored the design of a SI-BCI system, they compared different features and classifiers on data from nine subjects and their results revealed that linear classifiers and Filter Bank Common Spatial Patterns (FSCSP) were the most appropriate for SI-BCI design. In [9] a procedure of design of a SI-BCI classification stage built with an ensemble of classifiers derived from spatial and temporal filters specific for each subject was presented. The authors showed that by taking advantage of a large dataset from a high number of subjects, it is possible to classify the data of new subjects with accuracies between 64.3 and 74.6%, which are similar to those achieved with techniques dependent of the subject specific data. In [10], a regularization scheme to obtain a better estimate of the covariance matrices used in Common Spatial Patterns (CSP) and Linear Discriminant Analysis (LDA) training by using covariance matrices from other subjects was reported. Their results showed that it is possible to train a BCI for a new user with less data from this user than the SD-BCI approach, hence reducing the calibration time.

However, the systems described above are not optimal for real-time tests, since the algorithms that are proposed imply a large computational cost and they would require a robust set of microprocessors to achieve the required performance. Moreover, they use between 32 and 64 electrodes which would imply longer time spent in channel preparation. It may also increase the cost of a BCI system and therefore it would be complicated to implement them in a clinical application. On the other hand, these papers show that there is common information between EEG signals from healthy subjects, but this has not been evaluated with EEG data from patients with motor disability. For example, in [11, 12] investigated the ability of stroke patients to use a BCI based on MI showing a performance comparable to healthy subjects (from 74% to 87%). However, to the authors’ knowledge, there are no reports about implementing a SI-BCI with the data of patients.

To use BCIs in clinical practice is not only necessary to develop complex algorithms to identify and classify the EEG signals, but it is also important to develop new methods to optimize the design process of the systems. A SI-BCI that requires a minimal number of electrodes and training sessions is desirable. The essential step in the design of SI-BCIs is feature extraction from the data of a group of subjects to complement the data of the target user. Typically, algorithms to select a subset of features for correctly classifying the user intent are applied. Another possibility is to find a subgroup of subjects that share common EEG features with the target user, for example, between subjects of the same gender. In this paper a proof-of-concept study is presented to show that it is possible to improve the performance of a SI-BCI using data from groups of subjects classified by gender. In order to accomplish this, we present a methodology used to design a gender-specific SI-BCI based on hand motor imagery with EEG signals from a group of healthy subjects. We compare the performance of the proposed SI-BCI design methodology with those achieved by both the classical SI-BCI methodology and a SD-BCI design. The gender-specific BCI would reduce the number of training sessions for the target user, and improve the performance of a BCI where a SI design is applied. Finally, the potential use of this methodology was evaluated in two patients with subcortical stroke. Some of the healthy subjects EEG data and an introduction to concepts used in this work were reported preliminary in [13].

## Methods

This study was planned using previous knowledge [6, 14], which details that power normalized to a reference interval (before the cue to start MI) decreases in the alpha (8–13 Hz) and beta band (14–30 Hz) when the subject performs real movement or motor imagery of his or her hands. These changes in power are recorded, mainly, in the central channels located above the sensory-motor cortex and are known as Event Related Desynchronization (ERD). SI-BCIs proposed in this paper are designed using Common Spatial Patterns (CSP) method. CSP is a type of spatial filter and is the reference method to SD-BCI designs based on MI. One of its features is to increase the separation between filtered signals of two classes, and so it can enhance the classification performance.

After EEG signals recording, the first preprocessing step for CSP calibration is frequency filtering, followed by an adaptively learned spatial filter, then by a log-variance feature extraction, and after that a classification step applied to the log-variance features. These steps are described in more detail below.

### Subjects

The proposal was evaluated with a control group composed of 32 healthy subjects between 21 and 30 years old, with mean of 25.9 ± 2.94 years, 50% female and 50% male, with incomplete or complete bachelor degrees. All were right-handed, with normal or corrected to normal vision, without previous psychological diseases or brain injuries. An expert in clinical electrophysiology discarded any abnormality in the EEG using a qualitative approach.

In order to evaluate the potential use of this approach for stroke patients, two patients, one of each gender, were selected as the stroke group. Demographic and clinical information is shown in Table 1. Both patients suffered subcortical stroke and the qualitative analysis of their EEG recordings did not reveal any paroxysmal or epileptic alterations. Both patients were right handed and with normal vision. When EEG data were recorded, they had weakness in the right thoracic limb.

**Table 1:** **Demographic and clinical characteristics of the stroke patients**

Patient	Gender	Age	Year since stroke	Hemisphere stroke	Injury location
1	Male	50	1	Left	Posterior limb of the left internal capsule
2	Female	57	3	Left	Left thalamic pulvinar nuclei with extension to the left internal capsule

All participants achieved a normal performance in the subscales of digit detection and visual detection of the neuropsychological test NEUROPSI Attention and Memory which evaluates the capacity to follow instructions and concentrate in repetitive tasks without being distracted by other stimuli [15]; this test was developed and standardized for spanish-speaking populations. Before the EEG recordings the participants signed an informed consent approved by the Ethics and Research Committee of the National Institute of Rehabilitation in Mexico.

### Experimental task

**Figure 1:**
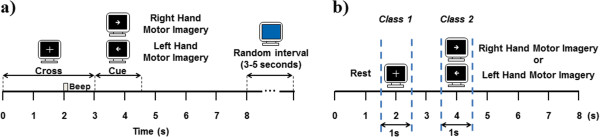
**Timing diagram of the experimental task and feature extraction. a)** Experimental paradigm used for the EEG recordings. At the white cross the subjects rested with their eyes opened, at the left arrow, subjects performed left hand motor imagery, and right hand motor imagery at the right arrow. At the blue background, subjects could blink and rest their eyes. **b)** Shows the time interval selected as class 1(REST from 1.5 s to 2.5 s) and class 2 (RIGHTMI or LEFTMI from 3.4 s to 4.5 s) for the calibration CSP and feature extraction stages.

None of the participants had previous knowledge of the experiment or training to perform motor imagery tasks, which where explained using verbal instructions minutes before initiating the EEG acquisition. Participants were seated on a comfortable armchair and a computer screen was placed in front of them at a distance of 1.50 m. Each EEG recording is composed of a series of trials with duration of 8 s that initiate with the presentation of a white cross in the center of a black background, followed by a short duration warning tone at 2 s. At the third second, the cross was replaced by an arrow pointing at either the left or the right side of the screen during 1.5 s. During the cross, subjects were instructed to stay in rest with their eyes open without performing motor tasks. When the arrow appeared subjects performed continuous movement of opening and closing the right or left hand depending on the arrow’s direction. Movement was performed until a blue background appeared without any visual guide, during a random interval between 3 and 5 s, in which they could blink their eyes. The experiment was based on Graz paradigm established by Pfurtscheller et al. [6]. A visual interface described in [16] was developed for the presentation of visual cues that indicate the participant when to perform the tasks. Timing diagram of the experiment can be seen in Figure 1a. The trial was repeated 20 times, the right and left arrow appeared randomly 10 times each to avoid the participants’ habituation. The average frequency for opening and closing the hand of all participants was of approximately 0.6 Hz. After performing the movement tasks, the subjects were asked to perform motor imagery tasks in which the instruction was to imagine the sensation of performing a same movement with either left or right hand. A run is made from 20 movement tasks and 20 MI tasks. This paper presents only the analysis for MI tasks.

In order to reduce the variability between trials in the SI-BCI design, a total of 30 healthy subjects performed only one run (10 trials per class). For two healthy subjects (one of each gender) EEG signals were recorded in two sessions of six runs each one (120 trials per class), the sessions were performed at 24 hours interval. The stroke group performed four sessions of three runs each one (120 trials per class), because of the limited availability of the patients the sessions were done at one week intervals.

### Signal acquisition

Eleven electrodes where placed in the subjects scalp, over sensory-motor cortex, according to the international 10–20 system (T3, P3, C3, Cz, C4, P4, T4, F3, Fz, F4 y Pz). Electrodes were also placed in the orbiculus oculi muscle on both eyes to record eye movements. EMG electrodes were placed in both arms (above the deep flexor and superficial muscles of the fingers) to verify that hand movements are present in the movement tasks, and the absence of them in the motor imagery tasks. For all recordings, electrode impedances were kept below 5 kΩ. EEG signals of group control were recorded with a Nicolet amplifier model NicONE with 32 channels and 16-bits resolution; ground and reference electrodes were located in the central forehead line. For stroke group signals were recorded with a g.TEC® amplifier, model gUSBamp with 16 channels and 24-bits resolution; ground was placed in the AFz position of the international 10–10 system and the reference electrode was located at the right ear lobe.

### Pre-processing

EEG signals in referential configuration were re-referencing using Common Average Reference (CAR). In CAR, the average value of the entire electrode montage (the common average) is subtracted from that of the channel of interest. Because it emphasizes components that are present in a large proportion of the electrode population, the CAR reduces such components. CAR provides an EEG recording that is nearly reference-free [17]. After that the re-referenced EEG signals were conditioned with a 30 Hz low-pass filter, an 8 Hz high-pass filter and two band-stop filters of 59–61 Hz and a 119–121 Hz, all of them were 20th order Butterworth type. The broad frequency band (which includes alpha and beta band) was chosen because it gives better classification results compared to narrow bands and is used successfully in the BCI based on CSP [18, 19].

All files were read, preprocessed, and processed using the Matlab® R2012b software from Mathworks® and the free license toolbox Fieldtrip [20], running on a computer Intel Pentium Core i7 with 12 GB RAM memory, a hard disk of 1 TB and Windows 8 operating system.

### Time-frequency analysis

A time-frequency analysis based on the complex Morlet wavelets [21] was performed to examine the significant ERD in the experimental conditions and select the time windows for feature extraction stage. All analyses were performed in the time interval from 1 s to 7 s of the run and in the frequency band from 8 Hz to 30 Hz using a resolution of 0.5 Hz. For each trial and each channel a Time-Frequency Representation (TFR) was calculated and normalized to a reference interval from 1.5 to 2.5 s. The reference interval, used to normalize the TFR was selected according to our previous results [22].

### CSP calibration

CSP finds a decomposition of the EEG recordings of two classes (calibration data) such that the variance for one class is maximized while the variance for the other class is minimized at the same time. The spatial patterns are determined by the simultaneous diagonalization of two covariance matrices, which is equivalent to the generalized eigenvalue problem. The first and the last component in the list are the first best patterns for discrimination between the two classes, and so on for the other components in the list. Typically 4 or 6 components in pattern space are sufficient for the discrimination task, so these patterns may be thought of as spatial filters that select the most relevant spatial aspects for the discrimination task. The detail procedure to calculate CSP is described in [23, 24].

In our case, CSP filters were calculated on 1-second time window for every task (see Figure 1b). Time window was selected according to the time-frequency analysis presented above. We only present results for the interval from 3.5 s to 4.5 s for right and left hand MI tasks and from 1.5 s to 2.5 s for the rest interval. We retain from 2 to 8 components to filter EEG channels to select the best option for the SI-BCI design. Here we present only the performances obtained using 8 spatial filters.

### Feature extraction

The features used for classification are obtained from the variances of the spatially filtered EEG channels. The feature vector for trial *i* is composed of the *2 m* variances (*m* is de number of selected components) of the p-row of the filtered EEG (*Z*), normalized by the total variance of the projections retained, and log-transformed (see Equation 1).
1

### Classification

The feature vectors from the calibration data are used to estimate the parameters of a linear classifier. An LDA classifier was selected, because it has proved to produce similar accuracy classification as other methods (including non-linear ones) but with lower computational cost [8, 25]. LDA was implemented by using the features obtained from several subjects as calibration data, which simulate data obtained from a single subject, as it is done in SD-BCI. To classify new data, we again obtain the feature vector with equation (1) for the new data (i.e. for the target user data) using the spatial filters obtained from the calibration data, and feed this feature vector into the classifier. Three configurations to calibrate CSP and compute log-variance features were implemented as follows: (1) BCI-All: using EEG signals from the 15 female and 15 male subjects from the group control, (2) BCI-Women: using data from 15 female subjects, (3) BCI-Men: using data from 15 male subjects.

The design of each classifier was divided in the three classical stages: training, validation and test. For the training stage the log-variance features of the selected subjects were used to train the LDA. In the validation stage a “leave-one-subject-out” method [9] was used, meaning that mixed data from all except one subject were used for the training of the classifier, whereas the data for the remaining subject were used to test the classifier. In other words, the classifier was tested with a subject whose training data did not include its own recordings, but did include those from other subjects. Finally, the test stage was performed using log variance-features from two healthy subjects (target users) who performed 240 trials and that were not included in CSP calibration stage (see Table 2 for details).

**Table 2:** **Number of trials of hand motor imagery collected for the experiments**

Stage	Subjects	Quantity	Trials per class	Total of trials per class
*Validation*	Male subjects	15	10	150
	Female subjects	15	10	150
*Test*	Male subject	1	120	120
	Female subject	1	120	120
	Male patient	1	120	120
	Female patient	1	120	120

Thirty healthy subjects were used in the SI-BCIs validation stage using the “leave-one-subject-out” method. Two other healthy subjects and two stroke patients were used for the test stage in the comparison of the performance of SI-BCIs and SD-BCIs.

The classification accuracy (ACC) was calculated to measure the BCIs performance. Classification was performed for right hand motor imagery (RIGHTMI) vs. rest (REST), left hand motor imagery (LEFTMI) vs. rest (REST), and for RIGHTMI vs LEFTMI.

To assess the implementation of a subject-independent design it is important to compare its performance against subject-dependent designs; for this reason two SD-BCIs were designed with the features from the two healthy subjects and they were compared with the performance of the BCI-All, BCI-Women, and BCI-Men. This was done by dividing the dataset from the target user into a calibration set, composed of 30 trials per class, for computing the CSP filters and a testing set, composed of the remaining 90 trials per class, for training and testing the LDA classifier. In order to validate and avoid trends in the training data pool, as well as the CSP and classifier overfitting, a 10x4-Fold cross-validation was used.

### Practical level of chance calculation

To be able to estimate the reliability of a gender-specific BCI approach, it is not enough to report classification accuracies. This is, because the chance level in a 2-class paradigm is not exactly 50%, but it is 50% with a confidence interval at certain level α depending on the number of trials on which the computations are based. The practical level of chance [26] provides a convenient tool to verify if a %ACC lies significantly above chance level.

In order to define if the designed BCIs performances were better than random, practical level of chance was calculated using a confidence level of 95% for all used schemes, SD-BCI (120 trials per class), BCI-Men and BCI-Women (150 trials per class), and BCI-All (300 trials per class). The levels of chance were then compared with the %ACC obtained in classification stage.

### Statistical analysis

The statistical unpaired-sample Student-*t* test with a confidence level of 95% was applied in order to determine if there was a statistically significant difference between the %ACC obtained from the BCI-All, and the %ACC achieved with the gender BCIs. The paired Student-*t* test was applied to determine if there was a statistically significant difference between the %ACC of the BCI-Women and BCI-Men with the BCI-ALL and with the SD-BCI, from both healthy subjects as well as for the stroke patients.

### Potential use for subcortical stroke patients

Since stroke patients in this work have sub-cortex injuries that do not affect other brain functions [27], the possibility to use the gender-specific SI-BCIs designed with data of healthy subjects to classify the data from the patients was raised. In order to do this, the same methodology for testing the SI-BCI designs (BCI-MEN, BCI-WOMEN and BCI-All), described in section “Classification” was applied to the data of the stroke patients. ACCs were obtained from SD-BCIs for each stroke patient and they were compared to the results obtained from the SI-BCIs. To validate the results a 10x4-Fold cross-validation was used.

## Results

**Figure 2:**
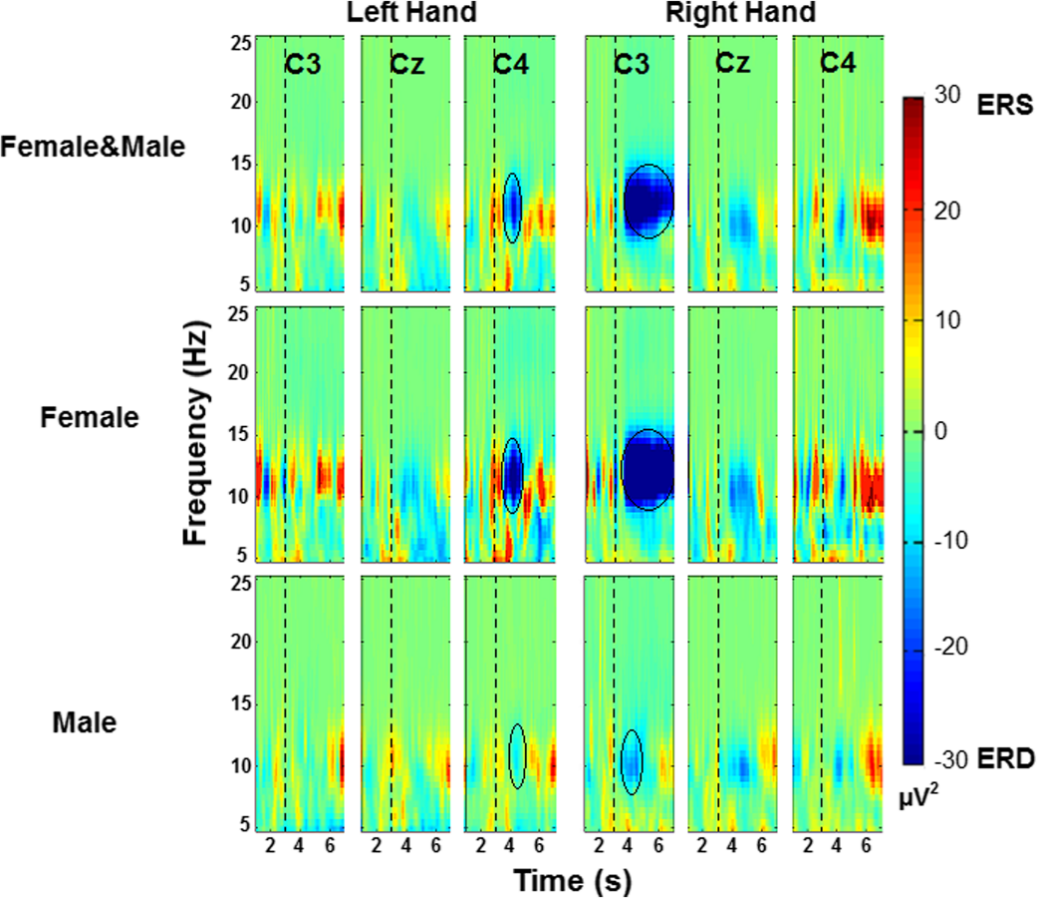
**Grand average TFRs in C3, Cz and C4 for hand motor imagery for all subject groups.** This figure shows the averaged TFRs across 30 participants, 15 female and 15 male subjects in 5 Hz to 25 Hz and from 1 s to 7 s. The dashed line at 3 seconds indicates motor imagery onset. ERD is shown in blue and ERS in red. The black circles highlight the maximum ERD generated.

**Figure 3:**
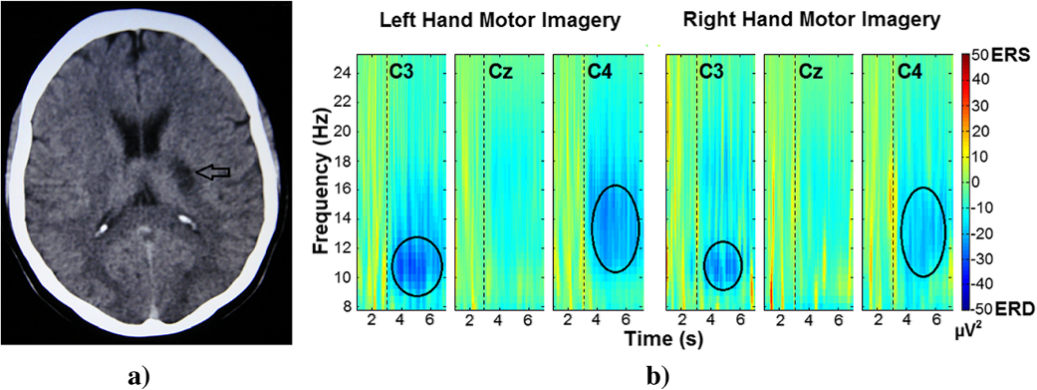
**Female patient CT image and averaged TFRs. a)** Representative image of the female patient’s CT (Computed Tomography). The black arrow indicates the location of the residual injury. **b)** Averaged TFR for left and right hand (affected limb) motor imagery in 8 Hz to 25 Hz from 1 s to 7 seconds. Dashed line at 3 seconds indicates the motor imagery onset. The black circles highlight the generated ERD.

**Figure 4:**
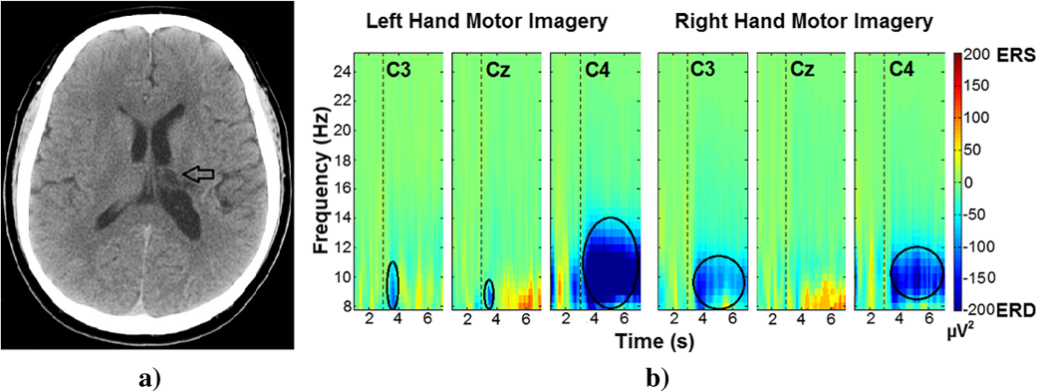
**Male patient CT image and averaged TFRs. a)** Representative image of the male patient’s CT (Computed Tomography). The black arrow indicates the location of the residual injury. **b)** Average TFR for left and right hand (affected limb) motor imagery in 8 Hz to 25 Hz from 1 s to 7 s. The dashed line at 3 seconds indicates the motor imagery onset. The black circles highlight the generated ERD.

Figure 2 shows the grand average maps for the 30 healthy subjects, 15 female and 15 male, TFRs are plotted for the left and right hand motor imagery. To visualize the event-related power changes, normalization with respect to the time interval from 1.5 s to 2.5 s was performed. TFRs show ERD, especially in alpha and beta frequency bands and in time windows from 3.5 s to 5.5 s. Figures 3 and 4 show the averaged TFRs for left and right hand MI trials performed by stroke patients of the three central channels (C3, Cz, and C4). As well as a CT where the brain’s damaged location is shown. The TFRs show that patients with subcortical stroke can generate ERD patterns similar to patterns of healthy subjects.

### Leave-one-subject-out validation results

**Figure 5:**
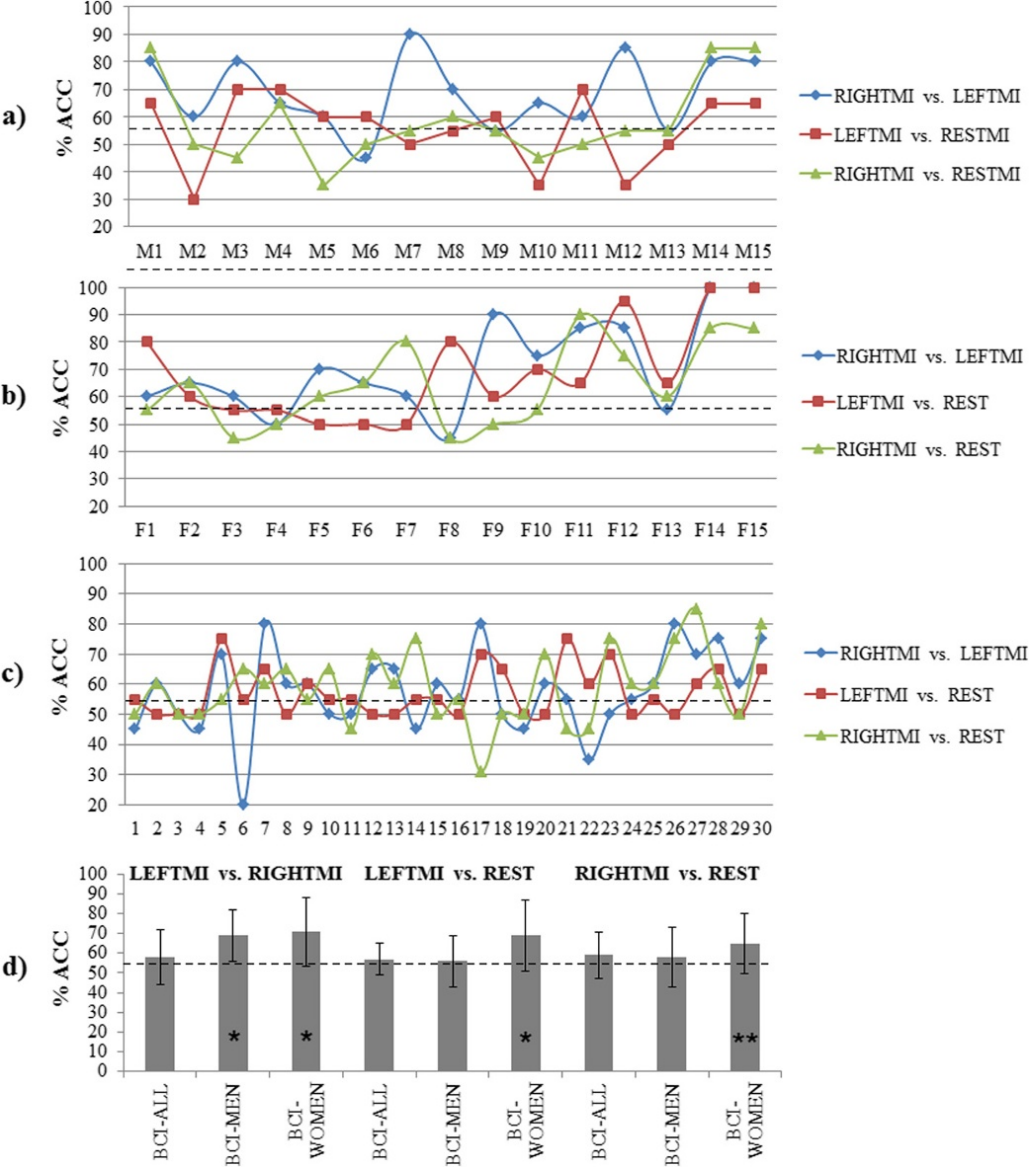
**Results of “leave-one-subject-out” method used for validation of SI classifiers.** This figure displays the validation results from the total of 30 subjects collected for SI-BCI designs. The performance of the classifiers is validated for the 3 tested conditions, this means, LEFTMI vs. RIGHTMI, LEFTMI vs. REST and RIGHTMI vs. REST. **a)** ACCs for the LDA classifier designed with data of 10 trials per class from 15 male subjects (BCI-Men). M1 to M15 represent which subject’s data is left out from the classifier training set. **b)** ACCs for the classifier assembled with data of 10 trials per class from 15 female subjects (BCI-Women). Similarly, F1 to F15 represent which female subject’s data is left out from the classifier training set. **c)** ACCs for the classifier assembled with data of 10 trials per class from all 30 subjects (BCI-All). Labels 1 to 15 are the male subjects and from labels 16 to 30 are the female subjects. **d)** Averaged performance for the validation stage of the proposed 3 classifiers (BCI-MEN, BCI-WOMEN and BCI-ALL) for all 3 tested conditions. The dashed line indicates the practical chance level. The asterisks (*p < 0.05 and **p < 0.1) indicate statistically significant difference between gender-specific BCIs and BCI-All.

Figure 5 shows the leave-one-subject-out cross-validation results for every configuration in the three analyzed conditions, LEFTMI vs. RIGHTMI, LEFTMI vs. REST, and RIGHTMI vs. REST. Figure 5a displays the %ACCs for all the 30 subjects for the BCI-All design. Similarly, Figure 5b shows accuracies for 15 male subjects for BCI-Men and Figure 5c for 15 female subjects for BCI-Women. In Figure 5d we average the %ACC for all subjects in every SI-BCI design to summarize the results. In LEFTMI vs. RIGHTMI condition, %ACCs for BCI-Women (71%) and BCI-Men (69%) are statistically significant greater (p < 0.05) than BCI-All (58%). In LEFTMI vs. REST only the %ACC in BCI-Women (69%) is statistically significant greater (p < 0.05) than BCI-All (57%). The same applies in the condition RIGHTMI vs. REST, where the performance of BCI-Woman (65%) is statistically significant greater (p < 0.1) than the performance of BCI-All (59%).

### Comparison between SI-BCIs and SD-BCIs designs

Table 3 displays the results for the test stage performed with two healthy subjects that were not included in calibration data, this means %ACC obtained for BCI-All, BCI-Men and BCI-Woman tested with data of the female and male subjects. The same table shows the results for the test of the designs with subcortical stroke patients’ data to show the potential use in this kind of users. In this table, it can be seen that for the male subject the performance of BCI-Men (60%) was statistically superior (p < 0.05) to BCI-All (55%) in the LEFTMI vs. REST condition. The other two conditions performances were near to the chance level for the three SI-BCI designs. For the female subject the performance of BCI-Women (79%) was statistically better (p < 0.05) than the performance of BCI-All (73%) in LEFTMI vs. REST condition, and the same applies for the RIGHTMI vs. REST condition in which the performance of BCI-Women (77%) was also better (p < 0.05) than that of BCI-All (73%). For the male patient in RIGHTMI vs. REST, the performance of BCI-Women (69%) was superior to BCI-All (64%). In LEFTMI vs. REST, the performance of BCI-Women (67%) was greater (p < 0.05) than BCI-All (61%), however for the RIGHTMI vs. LEFTMI, BCI-All (64%) performed similar that the gender-specific designs. For the female patient, for the RIGHTMI vs. LEFTMI condition BCI-All (64%) performed without significant differences with BCI-Men and BCI-Women. For the condition LEFTMI vs. REST, the BCI-Women (77%) performed significant better (p < 0.05) than BCI-All (74%) and, for RIGHTMI vs. REST, BCI-Men (83%) was the best.

**Table 3:** **Performance for the SI-BCIs designs in classifier test stage**

Target Subject	Type of SI-BCI design	LEFTMI vs RIGHTMI	LEFTMI VS REST	RIGTHMI VS REST
		Mean (SD)	Mean (SD)	Mean (SD)
*Male Subject*	BCI-Men	53 (5)	60 (3)*	54 (3)
	BCI-Women	54 (5)	58 (2)	58 (4)*
	BCI-All	**58 (2)**	55 (2)	55 (7)
*Female Subject*	BCI-Men	56 (2)	76 (1)	71 (3)
	BCI-Women	58 (4)	79 (3)*	77 (4)*
	BCI-All	**63 (6)**	73 (4)	73 (5)
*Male Patient*	BCI-Men	**65 (2)**	63 (1)	59 (2)
	BCI-Women	62 (2)	67 (3)*	69 (2)*
	BCI-All	64 (1)	61 (4)	64 (5)
*Female Patient*	BCI-Men	**63 (4)**	73 (3)	83 (7)*
	BCI-Women	59 (5)	77 (2)*	76 (3)
	BCI-All	62 (2)	74 (4)	76 (2)

This table shows the %ACCs from 2 healthy subjects and 2 stroke patients as target users. The mean and standard deviation (in parenthesis) was obtained for the 10×4-fold cross-validation for each tested condition. The best %ACC for each subject in every condition was marked in **BOLD**. The asterisk indicates the best gender-specific BCI design that was statistically significant better (*p < 0.05) than BCI-All.

Finally, Table 4 shows a comparison between the accuracies for SD-BCI (the traditional approach) designed for each healthy subject and stroke patients and the accuracies for the SI-BCI design with the best performance for that subject. Here it is seen that gender-specific BCIs (BCI-Men and BCI-Women) have better performance than BCI-All for most participants.

**Table 4:** **Performance of the subject-dependent BCI compared with of the best subject-independent BCI for each target user**

Target user	RIGHTMI vs LEFTMI			LEFTMI vs REST			RIGHTMI vs REST		
	SD-BCI	Best SI-BCI		SD-BCI	Best SI-BCI		SD-BCI	Best SI-BCI	
	Mean	Mean	BCI	Mean	Mean	BCI	Mean	Mean	BCI
*Male Subject*	61 (4)	58 (2)	All	59 (8)	60 (3)	Men	60 (4)	58 (4)	Women
*Female Subject*	61 (5)	63 (6)	All	81 (2)	79 (3)	Women	82 (3)*	77 (4)	Women
*Male Patient*	81 (4)*	65 (2)	Men	71 (8)	67 (3)	Women	78 (4)*	69 (2)	Women
*Female Patient*	62 (3)	63 (4)	Men	71 (6)	77 (2)	Women	73 (4)	83 (7)	Men

The SI-BCI with the significant better performance is shown in order to compare it against its SD-BCI counterpart, for all the 3 tested conditions (LEFTMI, RIGTHMI, and REST). The mean and standard deviation (in parenthesis) for the best SI-BCI was repeated of the %ACC showed in Table 3. The performance of the SD-BCI was statistically significant better (^*^p < 0.05) than the performance of SI-BCI only in the cases with an asterisk.

The calculated practical level of chance for BCI-All is at 54%, for BCI-Women and BCI-Men is at 55.5%, and for SD-BCIs is at 56.2%. %ACCs calculated are higher than these practical levels of chance.

## Discussion

The LDA classifiers for the proposed SI-BCI of this work that have been trained with a relatively small sample per subject can correctly differentiate with a %ACC above level of chance for the three tested conditions. It would be possible that by using a larger data sample for training the classifier, or by using other classification methods such as self-organizing maps or spiking neural networks, the performance of the classifiers may improve; however using these other approaches would mean a higher computational cost. In the validation stage of the SI-BCI designs (Figure 5d) BCI-Women and/or BCI-Men showed a better performance in all conditions than BCI-All, even though BCI-All was designed with a larger subjects’ sample than BCI-Women and BCI-Men, which could mean that gender allocation could improve the performance of a SI-BCI design. In the test stage, for eight conditions the gender-specific BCIs had better performances than the gender-non-specific one; for two other cases we have a similar performance between both types of SI-BCIs; and finally in only two test conditions the non-gender-specific BCI was better than the gender-specific one. When comparing the performance of designed SI-BCIs with SD-BCIs of both healthy subjects the results showed that the performances of the SI and SD designs were close to the chance level for RIGHTMI vs. LEFTMI; this could show that the EEG data in these subjects are so similar when performing hand MI that there is hardly any significant difference between them. This could be due to that all participants in this study were BCI illiterate or BCI naïve users. However for the RIGHTMI vs. REST and LEFTMI vs. REST, the performances of both the SI and SD designs were significant higher, so this could show that the subjects are performing motor imagery, as they generate different EEG patterns while they are in a rest condition and while performing MI. The better performance in these conditions could be due to better discrimination of the rest condition from MI, compared to discriminating left versus right hand MI.

On the other hand, the performance of the SD-BCI and SI-BCI designed for stroke patients in the three comparisons were superior to the level of chance (55.5%), and even reached accuracies above 80% in RIGHTMI vs. REST which corresponds to their affected limb. Table 4 shows that for nine out of twelve tested conditions the gender-specific SI designs performances were equivalent to the SD ones. These results would imply that it is possible to design spatial filters using healthy subjects’ data to develop a SI-BCI system for patients with subcortical stroke, in order to reduce their training sessions.

The %ACCs obtained with the SI-BCIs were superior to level of chance and these results were obtained by training a classifier only with the data of 11 EEG channels, so the computational cost of implementing a SI-BCI system would be less than the cost of other systems with a better performance but with the limitation of having to process data from 32 to 64 channels.

Results show that it is not always true that a SI-BCI trained with the same gender as the target user may have a better performance than one trained with the opposite gender, since the SI-BCI designs allocated by gender showed mixed results in these cases. The results of this study may evidence that common EEG power features exist between some subjects, and that this common power features are more likely to be useful in SI-BCI designs if the system is designed by taking into account the gender of the training sample. It is possible that using data from other subjects could potentially increase the performance of a BCI system and reduce training and calibration sessions, which is especially useful when the target user has a motor disability.

## Conclusions

There is still a long road ahead for having a fully functional BCI for controlling orthosis, prosthesis, or wheelchairs, which may prove usefulness for disabled people, such as stroke patients. In the current literature many research has focused in improving the BCI performance, but it is not only necessary to develop more complex algorithms to classify EEG features, it is also important to establish new methodologies in the design process by taking into account the electrical brain and physical characteristics of patients with motor disabilities. As an example, in this paper we have proposed a methodology to improve the performance of SI-BCIs considering the subjects gender.

Our results show that the SI gender-specific designs still have to be tested with data from more subjects in order to make a clear statement that a SI gender-specific design will be better than one that is not, however the results obtained in this study for the validation stage indicate that this approach could be promising to improve the performance of SI-BCI designs.

The results of the tests with two subcortical stroke patients suggest that these patients generate ERD patterns similar to those of healthy subjects, therefore a SI-BCI design could be used in stroke patients, without the need of having an EEG data base of patients with this type of injuries. The importance of developing a simpler and accessible BCI for patients with motor disability caused by stroke goes beyond controlling an orthosis or another robotic device, since has been demonstrated that exercise and stimuli made when performing motor imagery, can also be an effective method, in its own means, for the neuro-rehabilitation of these patients [28, 29].

As a future work, the implementation of an algorithm, as FBCSP [7], that improves the %ACC is aimed. By having a larger sample of patients and healthy subjects, we could increase the chances to be able to find better matching log-variance features for the BCI user and thus increase the BCI design performance.

## References

[1] Friedrich EV, Scherer R, Neuper C (2013). Long-term evaluation of 4-class imagery-based brain-computer interface. Clin Neurophysiol.

[2] Hazrati MK, Erfanian A (2010). An online EEG-based brain-computer interface for controlling hand grasp using an adaptive probabilistic neural network. Med Eng Phys.

[3] Kus R, Valbuena D, Malechka T, Graeser A, Durka P (2013). Asynchronous BCI based on motor imagery with automated calibration and neurofeedback training. IEEE Trans Neural Syst Rehabil Eng.

[4] Chih-Wei C, Chou-Ching KL, Ming-Shaung J (2009). Hand orthosis controlled using brain-computer interface. J Med Biol Eng.

[5] Wolpaw JR, Birbaumer N, McFarland DJ, Pfurtscheller G (2002). Brain-computer interfaces for communication and control. Clin Neurophysiol.

[6] Pfurtscheller G, Neuper C (2001). Motor imagery and direct brain-computer communication. Proc IEEE.

[7] Ang KK, Chin ZY, Wang C, Guan C, Zhang H (2012). Filter bank common spatial pattern algorithm on BCI competition IV datasets 2a and 2b. Front Neurosci.

[8] Lotte F, Guan C, Ang KK (2009). Comparison of Designs Towards a Subject-Independent Brain-Computer Interface based on Motor Imagery. Proceedings of the 31st IEEE EMBC.

[9] Fazli S, Popescu F, Danóczy M, Blankertz B, Müller K-R, Grozea C (2009). Subject-independent mental state classification in single trials. Neural Netw.

[10] Lotte F, Guan C (2010). Learning from Other Subjects Helps Reducing Brain-Computer Interface Calibration Time. Proceedings of the IEEE ICASSP.

[11] Ang KK, Guan C, Chua KSG, Ang BT, Kuah CW, Wang C, Phua KS, Chin ZY, Zhang H (2011). A large clinical study on the ability of stroke patients to Use an EEG-based motor imagery brain computer interface. Clin EEG Neurosci.

[12] Prasad G, Herman P, Coyle D, McDonough S, Crosbie J (2010). Applying a brain-computer interface to support motor imagery practice in people with stroke for upper limb recovery: a feasibility study. J Neuroeng Rehabil.

[13] Cantillo-Negrete J, Gutierrez-Martinez J, Carino-Escobar RI, Flores-Rodriguez TB, Elias-Vinas D (2013). Time-Frequency Analysis of EEG Signals from Healthy Subjects Allocated by Gender for a Subject-Independent BCI-Based on Motor Imagery. 6th International IEEE/EMBS Conference on Neural Engineering (NER): 6–8 November 2013; San Diego CA.

[14] Birbaumer N (2006). Breaking the silence: Brain-computer interfaces (BCI) for communication and motor control. Psychophysiology.

[15] Ostrosky-Solis F, Gómez-Pérez E, Ardila A, Rosselli M, Matute E, Pineda D, Lopera F (2003). Batería Neuropsicológica NEUROPSI Atención y Memoria, 6 a 85 años de edad.

[16] Cantillo-Negrete J, Gutiérrez-Martínez J, Cariño-Escobar RI, Elías-Viñas D (2013). Module to Present and Identify Motor Imagery Tasks in Electroencephalography. Proccedings of the VIII Pan American Health Care Exchanges Conference (PAHCE):29 April-04 May 2013.

[17] Bertrand O, Perrin F, Pernier J (1985). A theoretical justification of the average reference in topographic evoked potential studies. Electroenc Clin Neurophysiol.

[18] Guger C, Ramoser H, Pfurtscheller G (2000). Real-time EEG analysis with subject-specific spatial patterns for a brain-computer interface (BCI). IEEE Trans Rehab Eng.

[19] Pfurtscheller G, Neuper C, Flotzinger D, Pregenzer M (1997). EEG-based discrimination between imagination of right and left hand movement. Electroenc Clin Neurophys.

[20] Oostenveld R, Fries P, Eric M, Schoffelen J-M (2011). FieldTrip: open source software for advanced analysis of MEG, EEG, and invasive electrophysiological data. Comput Intell Neurosci.

[21] Tallon-Baudry C, Bertrand O, Delpuech C, Pernier J (1997). Oscillatory gamma-band (30–70 Hz) activity induced by a visual search task in humans. J Neurosci.

[22] Cantillo-Negrete J, Gutiérrez-Martínez J, Flores-Rodríguez TB, Cariño-Escobar RI, Elías-Viñas D (2014). Characterization of electrical brain activity related to hand motor imagery in healthy subjects. Rev Invest Clin.

[23] Müller-Gerkinga J, Pfurtscheller G, Flyvbjergc H (1999). Designing optimal spatial filters for single-trial EEG classification in a movement task. Clin Neurophysiol.

[24] Ramoser H, Muller-Gerking J, Pfurtscheller G (2000). Optimal spatial filtering of single trial EEG during imagined hand movement. IEEE Trans Rehabil Eng.

[25] Bashashati A, Fatourechi M, Ward RK, Birch GE (2007). A survey of signal processing algorithms in brain-computer interfaces based on electrical brain signals. J Neural Eng.

[26] Müller-Putz JR, Scherer R, Brunner C, Leeb R, Pfurtscheller G (2008). Better than random? A closer look on BCI results. Int J Bioelectromagn.

[27] Sharma N, Simmons LH, Jones PS, Day DJ, Carpenter TA, Pomeroy VM, Warburton EA, Baron JC (2009). **Motor imagery after subcortical stroke: A Functional Magnetic Resonance Imaging study**. Stroke.

[28] García Carrasco D, Aboitiz Cantalapiedra J: **Efectividad de la imaginería o practica mental en la recuperación funcional tras el ictus: revisión sistemática.** *Neurologia* ᅟ, **ᅟ:**ᅟ. in press

[29] Dickstein R, Deutsch JE, Yoeli Y, Kafri M, Falash F, Dunsky A, Eshet A, Alexander N (2013). Effects of integrated motor imagery practice on gait of individuals with chronic stroke: a half-crossover randomized study. Arch Phys Med Rehabil.

